# A Phase II Trial of Perioperative Oral Itraconazole for the Management of Low‐Risk Basal Cell Carcinoma

**DOI:** 10.1111/exd.70264

**Published:** 2026-05-08

**Authors:** Rodrigo Pérez Pereira, Dienifer Hermann Sirena, Sérgio Jobim de Azevedo, Monique Maria Franco da Silva, Mariani Magnus da Luz Andrade, Charles Francisco Ferreira, Tiago Elias Heinen, Renato Marchiori Bakos

**Affiliations:** ^1^ Postgraduate Program in Medical Sciences Universidade Federal Do Rio Grande Do Sul Porto Alegre RS Brazil; ^2^ Clinical Research in Oncology (UPCO) Hospital de Clínicas de Porto Alegre Porto Alegre RS Brazil; ^3^ Postgraduate Program in Physiology, Institute of Basic Health Sciences Universidade Federal Do Rio Grande Do Sul Porto Alegre RS Brazil; ^4^ Faculty of Medicine Universidade Federal Do Rio Grande Do Sul Porto Alegre RS Brazil; ^5^ Department of Dermatology Hospital De Clínicas De Porto Alegre Porto Alegre RS Brazil

**Keywords:** angiogenesis, antineoplastic agents, cancer, carcinoma, hedgehog proteins, neoadjuvant therapy, skin neoplasms

## Abstract

Recently, new treatment strategies have been developed for advanced or metastatic basal cell carcinoma (BCC), including Hedgehog pathway inhibitors. Itraconazole has demonstrated clinical activity in such cases by blocking the smoothened (SMO) receptor. Such a strategy could be used in early disease. The objective of this Phase II study was to evaluate the clinical and molecular efficacy of Itraconazole in low‐risk BCC patients who were primarily candidates for surgical excision. Lesions were assessed according to RECIST 1.1 criteria, with target lesions being at least 10 mm in size after confirmatory biopsy. Patients received itraconazole tablets 200 mg twice daily for 60 days before resection. The median tumour diameter before treatment was 14 mm (IQR 11–16 mm), and after treatment, 13 mm (IQR 11–15 mm), and this reduction in tumour size was statistically significant (*p* < 0.0001). Coupled with the decrease in tumour size, a decrease in the expression of CD105, an endothelial marker (*p* < 0.0001), was observed. In conclusion, neoadjuvant itraconazole for 2 months was able to reduce the diameter of low‐risk BCC, and the effect was associated with a decrease in tumour angiogenesis and a favourable safety profile.

## Graphical abstract

Neoadjuvant treatment with oral itraconazole for two months demonstrated significant responses in low‐risk basal cell carcinomas. This effect was associated with decreased tumour angiogenesis, presenting a favourable safety profile for patients.

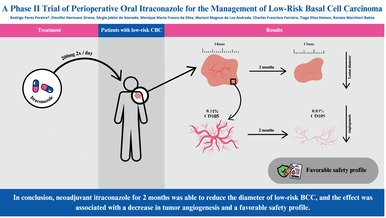

**Abbreviations**
ALT: alanine aminotransferase
AST: (aspartate aminotransferase)
BCC: basal cell carcinoma
GLI: Glioblastoma family transcription factors
HBP: high blood pressure
HH: hedgehog
IQR: interquartile range
Ki67: (protein nuclear Ki‐67)
NCI‐CTC: National Cancer Institute Common Toxicity Criteria
PT: prothrombin time
PTCH: protein patched homologue
RECIST: Response Evaluation Criteria in Solid Tumours
SMO: Smoothened
TGF: Transforming Growth Factor
US: United States
VEGFR2: Vascular endothelial growth factor receptor 2

## 1 Introduction

Non‐melanoma skin tumours are the most common neoplasms in humans, with basal cell carcinomas (BCC) representing around 70% of tumour incidence in this group [1]. The incidence of BCC has been increasing in the United States (US) and globally, likely due to greater ultraviolet radiation exposure and an ageing population. Approximately 2.8 million cases are diagnosed annually in the US, corresponding to over 3000 deaths per year [2]. However, this number may be underestimated as BCC is an indolent tumour and often goes undiagnosed [3]. BCC can be categorised as either localised (low‐risk) or advanced (high‐risk) to better define therapeutic strategies [4]. Treatment of localised BCC is primarily surgical, with high rates of local control and cure with complete excision of the tumour [5]. For advanced or high‐risk disease, including locally advanced and metastatic BCC, new treatment strategies have been developed. The prevalence of advanced or metastatic disease remains low, with only around 0.8% of all BCC being considered locally advanced. The prevalence of metastatic BCC is exceedingly low, with reported rates usually below 0.1%, depending on the source and the population studied and according to both historical data and systematic reviews [6, 7, 8]. The significant absolute number of high‐risk BCC cases results from the condition's high overall prevalence.

Recent advances in tumour biology knowledge have opened new therapeutic options for advanced or metastatic BCC. BCC arises from a cascade of events involving tumour suppressor genes and proto‐oncogenes, particularly the binding of Sonic Hedgehog (SHH) to the protein patched homologue (PTCH) receptor. This suppresses PTCH, activating the smoothened (SMO) receptor and Glioblastoma family transcription factors (GLI), which drive cellular proliferation. Around 90% of all BCCs have mutations that hyperactivate the Hedgehog (HH) pathway [9, 10].

The discovery of SMO's oncogenic role led to the development of SMO inhibitors like Vismodegib and Sonidegib, approved for treating advanced BCC [11, 12]. In the VISMONEO study, Vismodegib (150 mg daily for 4–10 months) improved surgical conditions in 80% of patients and led to a 71% overall response rate. This perioperative approach is crucial for improving surgical outcomes, particularly in head and neck tumours [13].

Additionally, the antifungal Itraconazole has shown potential as an HH pathway inhibitor. In vitro, it inhibited HH signalling even in SMO‐resistant cells [14]. A Phase II study of Itraconazole in advanced, non‐metastatic BCC patients found reduced cellular proliferation, measured through the cell activity marker Ki67 (nuclear protein Ki‐67), and decreased HH pathway activity [15]. These results suggest Itraconazole could be a more accessible treatment option with fewer side effects than current SMO inhibitors. This phase II trial aims to assess the clinical and molecular efficacy of Itraconazole for treating BCC in patients withlocalised, low‐risk disease who are primarily candidates for surgery.

## 2 Methods

### 2.1 Study Design

This is an open‐label, single‐centre phase II clinical trial involving patients from the Dermatology Service at the Hospital de Clínicas de Porto Alegre (HCPA) in the state of Rio Grande do Sul, Brazil.

### 2.2 Participants

Patients with a pathologically confirmed diagnosis of BCC who are followed at the Dermatology, Oncologic Surgery or Clinical Oncology outpatient clinics of HCPA were recruited. The diagnosis of BCC was defined by histopathological examination according to the World Health Organization (WHO) tumour classification criteria.

The sample size calculation for this phase II clinical trial was based on the two‐stage design proposed by Simon (1989) [16]. This optimal two‐stage design seeks to minimise patient exposure to ineffective treatment. In the first stage, 13 patients were enrolled and continuation to the second stage required observing at least one clinical response (partial or complete) among these 13 patients, based on RECIST 1.1 criteria. This threshold was met, and the study proceeded to enrol a total of 26 patients. The design assumed a response rate of ≤ 5% as uninteresting and ≥ 20% as desirable, with a one‐sided alpha of 0.05 and power of 80%.

This study included patients with a defined curative treatment plan who agreed to undergo experimental treatment for 60 days until the planned date of the definitive surgical procedure. Patients over 18 years old, with a performance status between 0 and 3, and at least one BCC lesion with a minimum diameter of 10 mm were included. Key exclusion criteria included chronic renal insufficiency stage, decompensated acute or chronic liver disease, symptomatic heart failure or ejection fraction less than 50%, use of other medications that could interfere with Itraconazole activity or metabolism, history of intolerance to Itraconazole, another malignant neoplastic disease under treatment or treated in the last year.

The study was conducted in accordance with the principles of the Declaration of Helsinki and Good Clinical Practice guidelines as defined by the International Conference on Harmonisation. The protocol was approved by the Research Ethics Committee of the Hospital de Clínicas de Porto Alegre in Plataforma Brasil under number CAAE 58687422.7.0000.5327 and registered in Clinical Trials under number NCT03972748. All patients provided written informed consent before enrollment.

### 2.3 Treatment

Patients received 200 mg Itraconazole tablets orally twice daily (12‐h interval between doses) for at least 60 days or until the date of surgery. This dose schedule was based on previous clinical data of Itraconazole use in patients with BCC [15]. This protocol did not include dose adjustments based on toxicity. Patients who experienced severe adverse reactions or those that affected their quality of life, which could not be managed otherwise, were to discontinue treatment.

### 2.4 Assessments, Toxicity and Follow‐Up

The patient's sample was characterised by age, sex, race, and presence of comorbidities such as smoking, diabetes, high blood pressure (HBP), alcoholism, obesity, and depression. All patients were assessed at baseline with a complete medical history, including general physical and dermatological examination. Patients with suspected or confirmed metastases should undergo staging imaging exams as indicated, and were excluded from the trial.

The histological type of BCC, location, measurement, and photographic documentation of target lesions were verified before and after the treatment period with itraconazole. In addition, abdominal ultrasound and chest X‐ray within 45 days before the start of the study), laboratory tests to check liver and kidney function (urea, creatinine, total bilirubin, AST (aspartate aminotransferase), ALT (alanine aminotransferase), PT (prothrombin time), and echocardiogram (only for patients with suggestive symptoms or established risk factors) were performed.

All patients were monitored and reassessed throughout the treatment period, and adverse effects of treatment were graded according to the National Cancer Institute Common Toxicity Criteria (NCI‐CTC 4) at each visit and up to 30 days after the last dose of treatment.

### 2.5 Clinical Evaluation of Response

All patients had their disease assessed according to Response Evaluation Criteria in Solid Tumours (RECIST) 1.1 criteria. For this method, target cutaneous lesions are those with at least 10 mm in the largest diameter. Dermoscopic measurements and clinical photographs were performed by a single investigator using standardised procedures, non‐blinded to treatment timepoints, during routine protocol visits. These measurements were used to define response criteria. Dermoscopic images were captured using a polarised light device (Dermlite DL4; 3Gen, Aliso Viejo, CA, USA) coupled to a digital camera system. Tumour measurements were performed using calibrated rulers to ensure consistency.

According to re‐evaluations during and at the end of treatment, patients were classified into the following response groups:

- Complete Response: Complete disappearance of the target lesion.
- Partial Response: Reduction of at least 30% in the largest diameter of the target lesion, compared to the baseline value.
- Stable disease: Patients who did not meet criteria for disease progression or response, either complete or partial.
- Disease progression: An increase of 20% in the largest diameter of the target lesion, compared to the baseline value.

### 2.6 Molecular Evaluation of Response

Target lesions were defined as measurable BCC lesions with a diameter of at least 10 mm after confirmatory biopsy. These lesions were measured and photographed according to standard procedures. Biopsies were performed using a dermatological punch for molecular marker assessment of HH pathway activation.

After inclusion in paraffin blocks, samples were sectioned by microtome to 3 μm thick slices. The obtained slices were placed on silanised slides, dried at 72°C for 2 h, and deparaffinised in xylol. Antigen recovery was performed by incubation with EnVision Flex Target retrieval solution (Agilent Technologies) at 95°C for 60 min. Endogenous peroxidase activity was blocked by setting samples in EnVision Flex Peroxidase‐blocking reagent (Agilent Technologies) for 5 min. The slides were incubated overnight with the following antibodies: Anti‐Gli3 antibody (ABCAM) and Anti‐CD105 antibody (ABCAM) using dilutions of 1:100 and 1:500, respectively. The reaction was visualised by incubating slides with EnVision FLEX/HRP (Agilent Technologies) for 20 min, followed by EnVision FLEX DAB+Chromogen (Agilent Technologies) and EnVision FLEX Substrate Buffer (Agilent Technologies) applications, according to the manufacturer's recommendations. Wash steps were performed by using EnVision FLEX Wash Buffer (Agilent Technologies). Aftervisualisation, the slides were counterstained with Harris haematoxylin for 20 s, differentiated in 2% ammonia water for 20 s, and then dehydrated in absolute ethanol. The stained slides were subsequently immersed in xylene and mounted with Entellan mounting medium (Sigma‐Aldrich).

Microscope slides containing the stained samples were evaluated by light microscopy (Olympus BX51), and four images of random fields were acquired with a microscope digital camera (Olympus DP71). The randomisation of fields included only areas with characteristic malignant cells, always considering the different subtypes of BCC. Analyses were performed using the ImageJ software. Only areas with BCC were selected, excluding healthy tissue or other adjacent structures. Following selection, the image was split into three colour channels (purple, red, and brown). The channel containing the stained colour (brown) had its colour threshold adjusted to a unique number, selecting only stained pixels. The final results were expressed in total area of selection and percentage of area occupied by marked pixels, indicating the tissue fraction with positive staining. All processes were executed with fixed parameters to ensure reproducibility, including scaling from pixels to μ m. Immunohistochemical analyses were performed by a single pathologist blinded to treatment timepoints.

### 2.7 Statistical Analysis

The data were entered into the SPSS program, version 18.0 [SPSS Inc., Released 2009. PASW Statistics for Windows, Version 18.0. Chicago: SPSS Inc.]. Descriptive analyses were performed using measures of central tendency and dispersion: For quantitative variables, means ± standard deviations (±SD) or medians (md) and interquartile ranges ([IQR], 25th and 75th percentiles) or 95% confidence intervals (CI), according to the Shapiro–Wilk normality test; for qualitative variables, absolute (*n*) and relative (%) frequencies were presented. Comparisons between the baseline and post‐treatment moments were performed using the Wilcoxon test, while analyses that considered the three follow‐up moments (baseline, during treatment, and post‐treatment) used the Friedman ANOVA test. For all analyses, the significance level adopted was 5%. Cohen's d was used to estimate the effect size of post‐treatment changes compared to baseline.

## 3 Results

### 3.1 Study Participants and Anatomopathological Analysis

During the period from 2019 to 2024, 41 patients were recruited, and 26 patients were included (8 patients refused participation, 5 were excluded based on laboratory results, 1 patient had a negative biopsy, and 1 patient had another concomitant neoplasia). The recruitment period was greatly affected by the COVID‐19 pandemic, which meant that for at least two years, most of our institution's resources had to be redirected to the management of COVID‐19 patients. According to data in Table 1, which presents the demographic and clinical characteristics of the 26 participants included in the study, the average age (±SD) of the individuals was 62.85 (± 11.61) years, ranging from 43 to 83 years, with the majority being female (61.5%) and of fair skin type (96.2%). These numbers are consistent with the expected demographic data for this population, where a higher incidence is observed in individuals with lighter skin, aged over 50 years, and a greater incidence in women [17, 18]. Of note, none of the patients in the cohort had obesity or a reported history of alcohol abuse. The data regarding tumours and histological subtypes of the participants included in the study can be found in Table 2. While literature reports typically describe superficial BCC as the second most frequent subtype, our data shows a higher‐than‐expected frequency of the infiltrative variant, which is considered to be more aggressive than the nodular or superficial subtypes [19, 20].

**TABLE 1:** Demographic and clinical characteristics of the study participants.

Variable	Total (*n* = 26)
Age (years)	62,85 ± 11,61
minimum – maximum	(43 – 83)
Gender	
Female	16 (61,5)
Male	10 (38,5)
Ethnicity	
White	25 (96,2)
Mixed	1 (3,8)
Sunscreen use	
No	3 (11,5)
Yes	1 (3,8)
INO	22 (84,6)
Smoking	
No	20 (76,9)
Yes	6 (23,1)
Diabetes	
No	24 (92,3)
Yes	2 (7,7)
Hypertension	
No	16 (61,5)
Yes	10 (38,5)
Alcohol abuse	
No	26 (100,0)
Yes	0 (0,0)
Obesity	
No	26 (100,0)
Yes	0 (0,0)
Depression	
No	23 (88,5)
Yes	3 (11,5)

*Note:* Data expressed as means ± standard deviations or absolute (*n*) and relative (%) frequencies.

Abbreviation: INO, information not obtained.

**TABLE 2:** Data regarding the tumours of the study participants.

Variable	Total (*n* = 26)
Subtype	
Superficial	2 (7,7)
Nodular	14 (53,8)
Infiltrative	9 (34,6)
NA	1 (3,8)
Location	
Head and Neck	4 (15,4)
Trunk	17 (65,4)
Members	5 (19,2)

*Note:* Data expressed as means ± standard deviations or absolute (*n*) and relative (%) frequencies.

Abbreviation: NA, not applicable.

Also, an apparent discrepancy was observed between the expected anatomical distribution of nodular BCCs and our findings. Although nodular BCC is typically described as more prevalent in the head and neck region, the majority of nodular lesions in our study were located on the trunk. This unexpected pattern may reflect selection bias, as patients enrolled were those referred for experimental treatment in a tertiary centre, which might not represent the general BCC population. Additionally, our small sample size may have amplified this variation. These findings highlight the importance of considering local epidemiological and referral patterns when interpreting anatomical distribution data in BCC studies.

### 3.2 Clinical Evaluation

**FIGURE 1:**
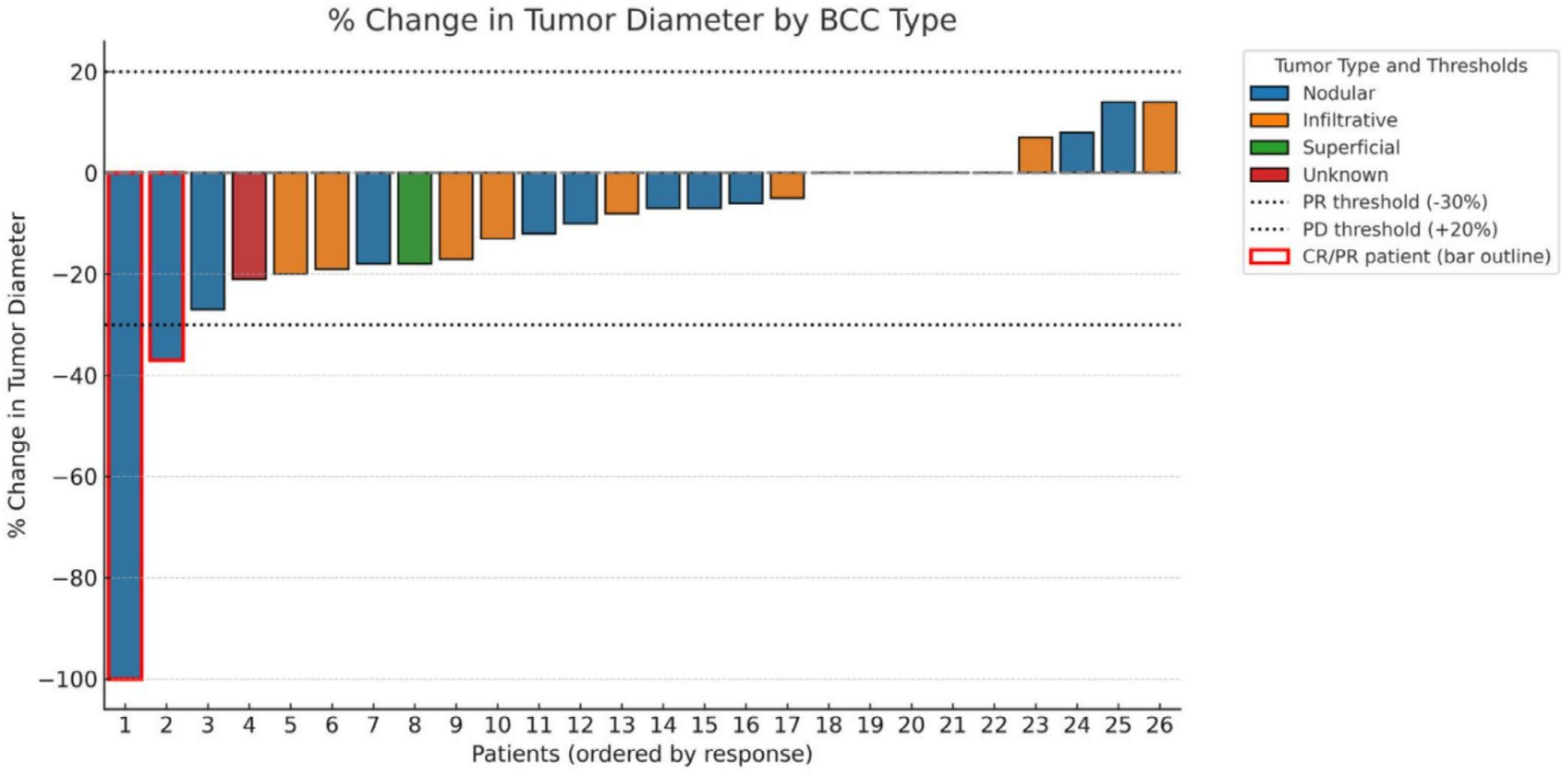
Waterfall plot depicting the individual percentage change in tumour diameter for all 26 patients treated with itraconazole for 60 days prior to surgical excision of low‐risk BCC. Each bar represents a single patient, ordered by magnitude of response. Bars are colour‐coded according to histologic subtype: Nodular (blue), infiltrative (orange), superficial (green) and unknown (red). Patients who achieved complete response (CR) or partial response (PR), as defined by RECIST 1.1 criteria, are outlined in red. Dashed lines indicate thresholds for partial response (−30%) and progressive disease (+20%).

**FIGURE 2:**
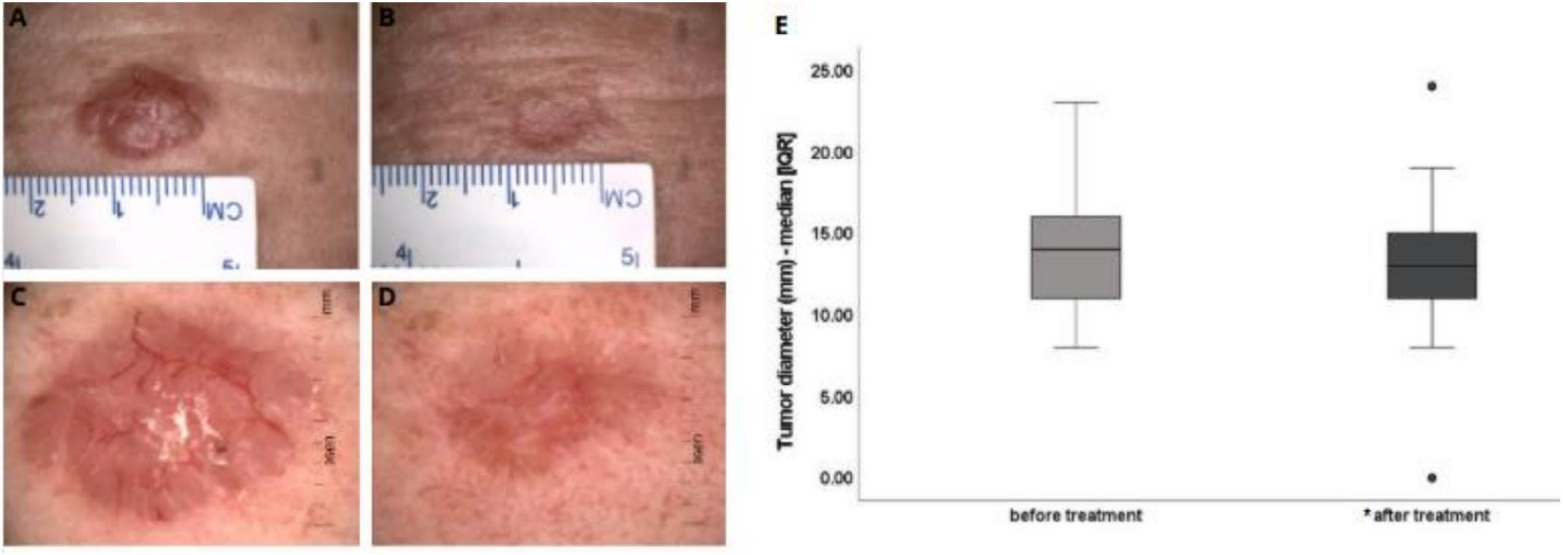
Clinical analyses of response to treatment. (A, B) Clinical images from the same patient showing (A) an erythematous telangiectatic irregular plaque, measuring 1.5 cm before treatment and (B) atrophic residual erythematous plaque, measuring approximately 1 cm after treatment; (C, D) Dermoscopic images showing (C) thick arborizing vessels and brown globules in the periphery before treatment and subsequently (D) thin and scattered vessels as well as shiny white structures (Original magnification × 20); (E) Data representing median tumour diameters before and after treatment, showing interquartile ranges (25th and 75th percentiles, [IQR]). Legend: Mm—millimetres. *Wilcoxon test, *p* ≤ 0.0001.

Regarding the overall response rate (ORR), 92.3% of patients had stable disease, 3.8% had a partial response, and 3.8% had a complete response (Figure 1). Tumour diameter before treatment ranged between 8 and 23 mm, with a median [IQR] of 14 [11–16] mm, and the diameter after treatment ranged between 0 and 24 mm, with a median [IQR] of 13 [11–15] mm, and this reduction was statistically significant (Wilcoxon test, *p* < 0.0001; Cohen's d = −0.15 [−0.69–0.40]). Tumour size reduction ranged from a maximum decrease of 8 mm to an increase of 3 mm, as can be seen in Figure 2.

### 3.3 Decrease in Angiogenesis Marker CD105 With Itraconazole Treatment

**FIGURE 3:**
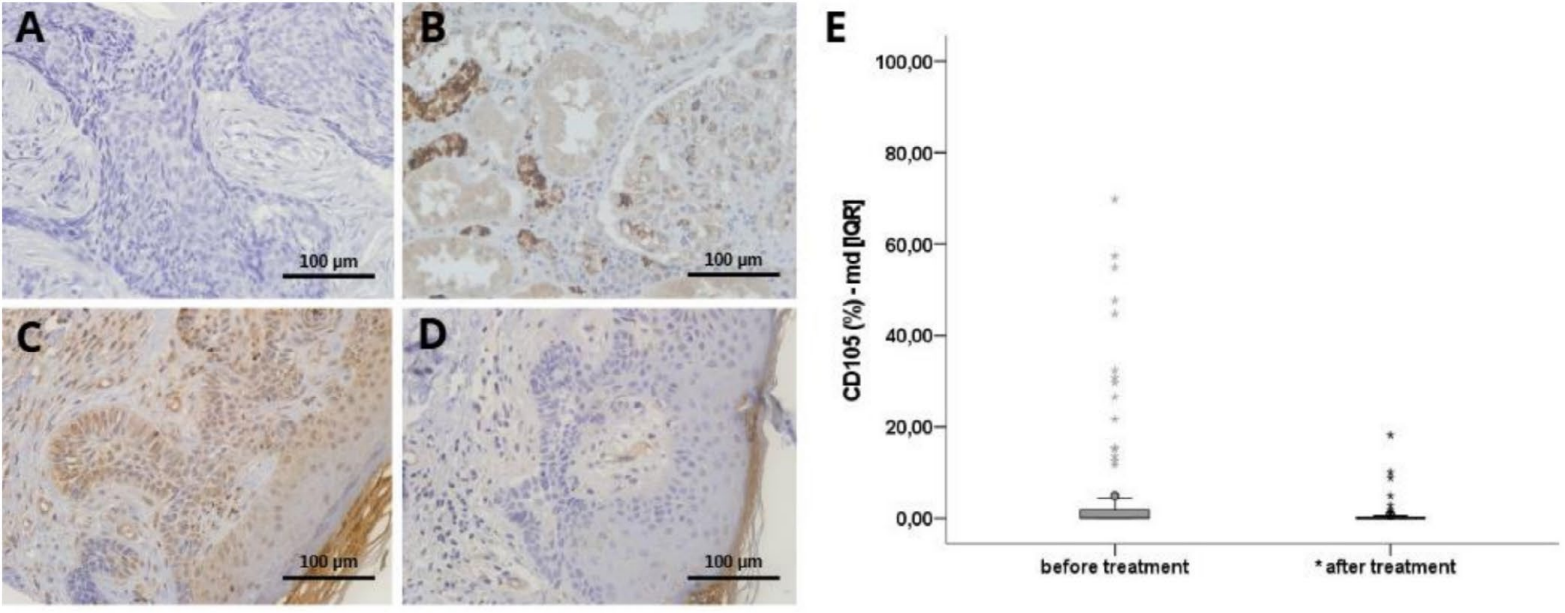
Molecular analysis of angiogenesis with CD105 by immunohistochemistry. (A) Negative control; (B) Positive control; (C) BCC before and (D) after itraconazole treatment, both images (C, D) corresponding to the same patient; (E) Data representing median values of CD105 staining before and after treatment, showing interquartile ranges (25th and 75th percentiles, [IQR]); sample size: 26 patients. Original magnification 400×. Legend: % per cent. *Wilcoxon test, *p* < 0.0001.

CD105, also known as Endoglin, is widely recognised as a reliable marker for endothelial cell proliferation, particularly related to angiogenesis in regenerating, inflamed tumours [20]. In our study, the percentage of stained cell area of Endoglin was diminished after treatment with Itraconazole, from 0.11 [0.01–1.86] to 0.03 [0.00–0.22], and this reduction was statistically significant (Wilcoxon test, *p* < 0.0001; Cohen's d = −0.48 [−1.03–0.07]), a result that reflects the antiangiogenic potential of the drug, supporting previous findings in the literature (Figure 3).

### 3.4 GLI3 Expression

**FIGURE 4:**
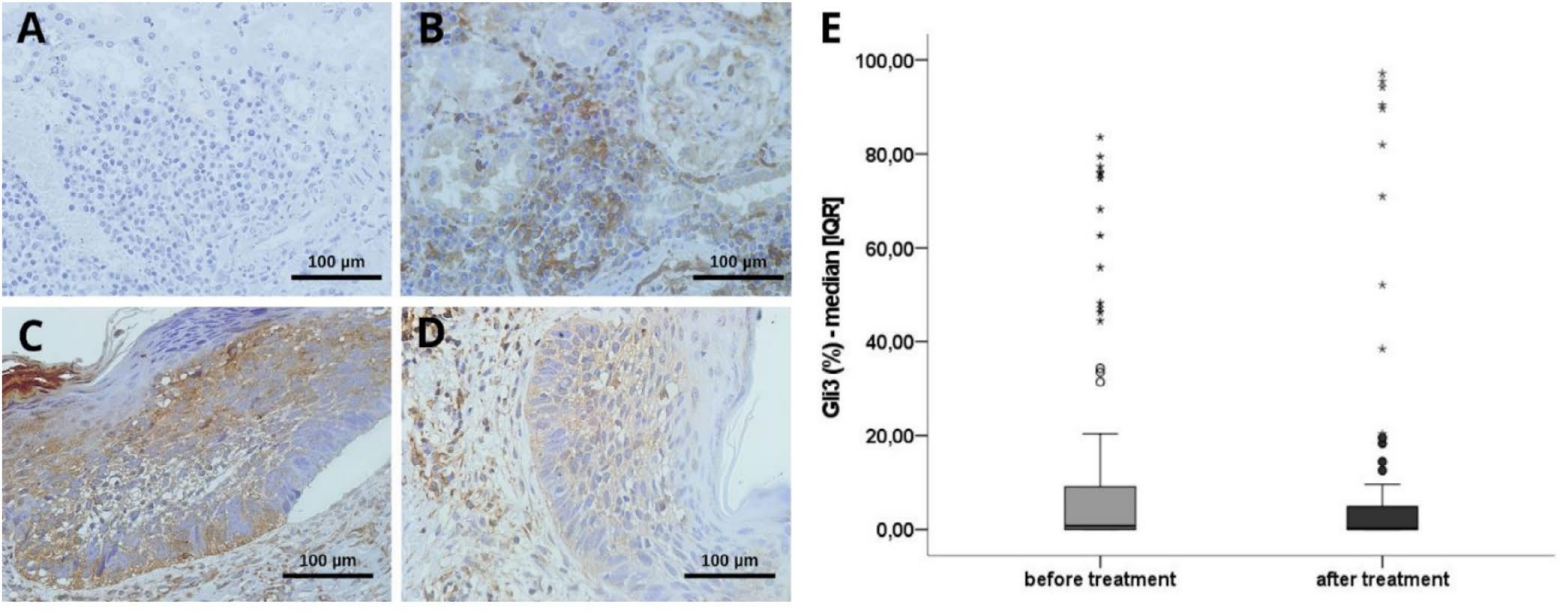
Molecular analysis of the Hedgehog pathway through GLI3 expression by immunohistochemistry. (A) Negative control; (B) Positive control; (C) Superficial subtype of BCC before and (D) after treatment with itraconazole, both images (C, D) corresponding to the same patient; (E) Data representing median values of GLI3 staining before and after treatment, showing interquartile ranges (25th and 75th percentiles, [IQR]); sample size: 26 patients. Original magnification 400×. Legend: % per cent. Wilcoxon test, *p* = 0.365.

GLI3 is a transcription factor that is part of the Hedgehog (HH) signalling pathway. Our results highlight the heterogeneity of tumoral GLI3 expression, with varying levels of intensity across different tumours [21]. While the differences in expression were not statistically significant due to sample variability, there appears to be a trend suggesting that Itraconazole treatment may reduce GLI3 expression (from 0.71 [0.03–9.17] to 0.33 [0.02–4.94]; Wilcoxon test, *p* = 0.365) (Figure 4).

Although Cohen's analysis indicated a small‐to‐medium effect size after treatment with itraconazole in both tumour diameter and Endoglin (tumour diameter: d = −0.15, 95% CI [−0.69 to 0.40]; CD105: d = −0.48, 95% CI [−1.03 to 0.07]), clinical improvement following itraconazole treatment could still be inferred.

### 3.5 Safety

The biochemical profile of the participants over time (baseline, during, and after treatment) can be seen in Table S1, which includes biochemical results related to renal and hepatic function that could change with the use of itraconazole. The initial median [IQR] values for urea (34 [26–39] mg/dL), creatinine (0.84 [0.74–1.01] mg/dL), total bilirubin (0.50 [0.40–0.80] mg/dL), AST (20 [17–23] U/L), ALT (19 [13–22] U/L), and the percentage activity of prothrombin time (100 [92–100]%) did not show statistically significant variations over time (Friedman ANOVA, *p* > 0.05 for all variables). From this perspective, we can observe that the use of itraconazole was safe, as no changes were observed during or after treatment. Such results are expected, as the safety of itraconazole is already well known [22].

## 4 Discussion

Our study demonstrated that the use of neoadjuvant itraconazole for 2 months was able to reduce the diameter of low‐risk BCC, and that effect was associated with a decrease in tumour angiogenesis and a favourable safety profile. The high rate of stable disease (92.3%) observed suggests that itraconazole may effectively suppress tumour progression in low‐risk BCC, even if objective responses are uncommon. While a statistically significant reduction in tumour diameter was achieved, the absolute reduction was modest (from 14 mm to 13 mm), and its clinical relevance in low‐risk tumours must be interpreted with caution. It is important to acknowledge that we utilised RECIST 1.1 criteria forstandardisation, although such unidimensional measurement may underestimate effects in irregularly shaped or superficial skin tumours compared to bidimensional (WHO) or volumetric assessments [23, 24]. Importantly, no patients experienced disease progression during the treatment period, and all went on to have their lesions surgically removed with clear margins, as per the initial plan. Notably, one minor protocol deviation occurred—a target lesion of 8 mm, which did not impact the overall conclusions.

This is the second trial to demonstrate clinical benefits of itraconazole in the treatment of BCC, an intervention that targets the HH signalling pathway, but the first one to clearly show a clinical antitumor effect possibly related to decreased angiogenesis in this context. The first published trial [15] involved patients with locally advanced BCC. In contrast, the current study focuses on low‐risk BCC tumours in patients who are candidates for primary surgical excision. These results are consistent with those seen with other HH pathway inhibitors, such as vismodegib and sonidegib, both Food and Drug Administration‐approved for locally advanced or metastatic BCC. While vismodegib and sonidegib have shown higher objective response rates (approximately 40%) in those scenarios, they are costly and frequently associated with adverse effects such as muscle cramps, dysgeusia and alopecia, which may limit adherence and long‐term use, especially if we consider the low‐risk context [9, 21]. In contrast, itraconazole is a widely available, low‐cost antifungal agent with a well‐established safety profile. Although its efficacy may be more modest, its favourable tolerability, oral administration, and affordability may position itraconazole as a valuable therapeutic option in real‐world BCC management—particularly in settings with limited access to newer targeted therapies. In the study by [25], itraconazole produced a 24% response rate in locally advanced patients, suggesting that while itraconazole is clinically useful, it may be less potent than vismodegib and sonidegib, which is supported by in vitro data [26].

Particularly, this study is the first to examine the effects of a longer treatment period (60 days) with itraconazole in patients with low‐risk BCC. The decision to use a 60‐day treatment period was based on previous research indicating that itraconazole requires sustained exposure to effectively inhibit the Hedgehog signalling pathway [25, 27].

We also evaluated the effects of itraconazole on biomarkers related to the HH pathway and angiogenesis, which were consistent with the expected biological activity of the drug. Angiogenesis, a hallmark of cancer development, plays a crucial role in supporting tumour growth and metastasis by supplying nutrients and oxygen; thus, reducing angiogenesis could be essential for improving the effectiveness of antineoplastic treatments [28]. Itraconazole may exert potential antiangiogenic effects by inhibiting endothelial cell proliferation and glycosylation of vascular endothelial growth factor receptor 2 (VEGFR2), which is crucial for the formation of new blood vessels [29]. Endoglin, or CD105, is widely recognised as a reliable marker for endothelial cell proliferation and angiogenesis in regenerating inflamed tumours [20, 30], and as a prognostic marker for recurrence in non‐melanoma skin cancers, where higher‐expressing tumours were more likely to recur [31]. While strategies targeting angiogenesis hold significant promise, current angiogenesis inhibitors have shown limited success, primarily demonstrating short‐term outcomes [32]. This highlights the gaps in our understanding and the pressing need for more effective therapeutic strategies, while also emphasising itraconazole's potential as a promising alternative for modulating angiogenesis in BCC.

The GLI family, GLI1, GLI2, and GLI3, plays key roles in embryonic development and is also implicated in carcinogenesis [19, 33]. While GLI1 (GLI family zinc finger 1) is a well‐established downstream effector of Hedgehog (HH) pathway activation and is commonly used as a readout of SMO inhibition [34], we made a deliberate decision to investigate GLI3 in this study. We sought to explore whether GLI3—which can act as both a transcriptional activator and repressor—might provide additional mechanistic insights into HH pathway modulation in BCC. Specifically, GLI3 upregulation has been linked to processes such as angiogenesis, malignant cell proliferation, and poor outcomes in cancers like squamous cell carcinoma, prostate, colorectal cancer, and lung adenocarcinoma [35, 36, 37, 38, 39, 40]. Conversely, the downregulation of GLI3 has been shown to induce chemotherapy resistance in acute myeloid leukaemia [41], underscoring the complex and context‐dependent role of GLI3 in cancer progression. In our study, we observed a trend toward reduced GLI3 expression after itraconazole treatment, which, although not statistically significant, holds potential interest for further investigations. Furthermore, given the non‐significant trend in reduced GLI3 expression, the observed antiangiogenic effects might also be mediated through HH‐independent pathways, such as the direct inhibition of VEGFR2 trafficking by itraconazole [29, 42].

Despite the extended treatment period, no severe adverse events were observed, and no patient had to discontinue treatment due to toxicity. This clinical scenario differs from that of patients with locally advanced or metastatic disease, as it involves a much lower tumour burden and likely less clonal variation [43].

Ultimately, therefore, it is worth noting that itraconazole is a drug that has been available for about 40 years [44], with a known safety profile, easy access to the general population, and has shown potential in phase II studies for the treatment and control of BCC [15]. Itraconazole emerges as a potential therapeutic agent for the treatment of low‐risk BCC and may be of interest, especially in scenarios in which the patient cannot undergo surgery in a timely fashion. In addition, there is an opportunity for the use of itraconazole in advanced scenarios for the control of inoperable or metastatic disease, especially when access to more modern HH inhibitors is difficult; a hypothesis to be studied in more robust studies.

Our study has important limitations. One of these limitations is the absence of a control group and the non‐blinding of the dermoscopic evaluation. Without a comparator arm, it is difficult to distinguish the observed tumour size reductions from potential confounding factors or natural fluctuations in tumour growth, particularly in indolent tumours such as low‐risk BCC. Although the statistically significant reductions in tumour diameter and molecular markers support the biological activity of itraconazole, the modest absolute change in size warrants cautious interpretation. Another such limitation is the small sample size, which may restrict the generalisation of our findings, and variability in tumour measurement techniques could influence the reported reductions in diameter. Future randomised controlled studies are needed to more definitively assess the clinical benefit of itraconazole in this setting.

## Author Contributions

Conceptualisation: R.P.P., D.H.S., R.M.B. Data Curation, Investigation and Project Administration: R.P.P., D.H.S. Methodology and Resources: R.P.P., D.H.S., M.M.F.S., M.M.L.A., C.F.F., T.E.H. Supervision: S.J.A., T.E.H., R.M.B. Writing – Preparation of original draft: R.P.P., D.H.S., M.M.F.S., T.E.H. Writing – Review and editing: R.P.P., D.H.S., S.J.A., M.M.F.S., C.F.F., T.E.H., R.M.B. All authors read and approved the final manuscript.

## Conflicts of Interest

The authors declare no conflicts of interest.

## Supporting information


**TABLE S1:** presents the results of the biochemical profile of the participants over time (baseline, during, and after treatment), which includes biochemical results related to renal and hepatic function that could change with the use of itraconazole.

## Data Availability

The data that support the findings of this study are available on request from the corresponding author. The data are not publicly available due to privacy or ethical restrictions.
