# Supplementary material for: A Phase II Trial of Perioperative Oral Itraconazole for the Management of Low‐Risk Basal Cell Carcinoma
Source: Exp Dermatol. 2026 May 8;35:e70264. doi: 10.1111/exd.70264 (PMC13155031; doi:10.1111/exd.70264)
Supplement: Supplementary file 1 — TABLE S1: presents the results of the biochemical profile of the participants over time (baseline, during, and after treatment), which includes biochemical results related to renal and hepatic function that could change with the use of itraconazole. [file EXD-35-e70264-s001.doc]

Supplementary Material
Supplementary Table 1. Biochemical profile of the study participants.


Variable
Total (N = 26)
	 *p-value

Baseline	During	Post-treatment		
Urea (mg/dL)	34 [26 – 39]

(minimum – maximum)	(16 – 82)	32 [29 – 36]

(20 – 59)	33,5 [28 – 41]

(21 – 60)	0,406	
Creatinine (mg/dL)	0,84 [0,74 – 1,01]

(minimum – maximum)	(0,63 – 1,50)	0,85 [0,74 – 0,94]

(0,55 – 1,33)	0,86 [0,76 – 0,91]

(0,61 – 1,33)	
0,853	
Total bilirubin (mg/dL)	0,50 [0,40 – 0,80]
(minimum – maximum)	(0,30 – 2,20)	0,50 [0,40 – 0,80]
(0,30 – 1,80)	0,45 [0,30 – 0,70]
(1,70 – 1,40)	
0,256	
AST (U/L) a	20 [17 – 23]
(minimum – maximum)	(14 – 64)	21 [18 – 28]
(12 – 82)	20 [18 – 29]
(18 – 98)	
0,927	
ALT (U/L)	19 [13 – 22]

(minimum – maximum)	(9 – 63)	22 [16 – 29]

(9 – 96)	21 [15 – 30]

(9 – 120)	
0,449	
Prothrombin Time (activity %)	100 [92 – 100]
(minimum – maximum)	(49 – 121)	100 [94 – 103]
(80 – 116)	99 [94 – 100]
(61 – 126)	
0,957	

Data expressed as medians and interquartile ranges ([IQR], 25th and 75th percentiles). Note: mg – milligram; dL – deciliter; L – liter; U – arbitrary unit; AST – aspartate aminotransferase; ALT – alanine aminotransferase; INO – information not obtained; p – statistical significance index. *Friedman ANOVA test. Significance set at 5%.
a N = 25 (96,2%). INO n = 1 (3,8%).
